# All-in-One Digital Microfluidics System for Molecular Diagnosis with Loop-Mediated Isothermal Amplification

**DOI:** 10.3390/bios12050324

**Published:** 2022-05-11

**Authors:** Siyi Hu, Yuhan Jie, Kai Jin, Yifan Zhang, Tianjie Guo, Qi Huang, Qian Mei, Fuqiang Ma, Hanbin Ma

**Affiliations:** 1 CAS Key Laboratory of Bio-Medical Diagnostics, Suzhou Institute of Biomedical Engineering and Technology, Chinese Academy of Sciences, Suzhou 215163, China; husiyi@sibet.ac.cn (S.H.); kai.jin@acxel.com (K.J.); zhangyf@sibet.ac.cn (Y.Z.); gtj19931101@163.com (T.G.); huangqi@sibet.ac.cn (Q.H.); qmei@sibet.ac.cn (Q.M.); 2 Guangdong ACXEL Micro & Nano Tech Co., Ltd., Foshan 528000, China; yuhan.jie@acxel.com

**Keywords:** electrowetting, digital microfluidics, all-in-one

## Abstract

In this study, an “all-in-one” digital microfluidics (DMF) system was developed for automatic and rapid molecular diagnosis and integrated with magnetic bead-based nucleic acid extraction, loop-mediated isothermal amplification (LAMP), and real-time optical signal monitoring. First, we performed on- and off-chip comparison experiments for the magnetic bead nucleic acid extraction module and LAMP amplification function. The extraction efficiency for the on-chip test was comparable to that of conventional off-chip methods. The processing time for the automatic on-chip workflow was only 23 min, which was less than that of the conventional methods of 28 min 45 s. Meanwhile, the number of samples used in on-chip experiments was significantly smaller than that used in off-chip experiments; only 5 µL of *E. coli* samples was required for nucleic acid extraction, and 1 µL of the nucleic acid template was needed for the amplification reaction. In addition, we selected SARS-CoV-2 nucleic acid reference materials for the nucleic acid detection experiment, demonstrating a limit of detection of 10 copies/µL. The proposed “all-in-one” DMF system provides an on-site “sample to answer” time of approximately 60 min, which can be a powerful tool for point-of-care molecular diagnostics.

## 1. Introduction

Nucleic acid analysis is a commonly applied molecular diagnostic technique with the advantages of good accuracy and high sensitivity, and it has been widely used in gene expression analysis, pathogen detection, disease diagnosis, and other fields [1,2]. However, traditional molecular laboratory analysis methods are usually complicated, time-consuming, and labor-intensive. In order to avoid aerosol contamination, the conventional molecular diagnostic laboratory is unexpectedly divided into three rooms—sample processing area, reagent preparation area, and testing area—and requires professional personnel to operate. Some of the better-funded organizations rely on sophisticated instruments and equipment, such as automatic pipetting workstations, which are large and can only be found in well-equipped hospital reference laboratories, which is not conducive to their further development and promotion. Therefore, especially in the face of the global epidemic outbreak and spread, the nucleic acid testing ranges from centralized structures in highly specialized laboratories, to diagnosis in close to patients, and even self-testing directly at home, the rapid, miniaturized, and convenient diagnostic equipment has become the trend [3,4].

Genetic testing usually involves these three steps: (1) DNA or RNA extraction from (complex) biological samples, which include the magnetic beads (MBs) method and centrifugal column method, and (2) Nucleic acid sequence amplification. The standard methods of nucleic acid amplification include polymerase chain reaction (PCR), loop-mediated isothermal amplification (LAMP), recombinase polymerase amplification (RPA), and nuclear acid sequence-based amplification (NASBA) [4,5,6]; (3) Signal detection of the amplification, usually through colorimetric, fluorescent signal detection or turbidity detection methods. Therefore, there is a need for a highly integrated system that includes nucleic acid extraction, amplification, and detection. To address this issue, many research groups used microfluidic technology to solve this problem [7]. However, most researchers mainly applied continuous or droplet microfluidic systems in the form of microchannels [8,9], which usually have complex surrounding mechanical pumps and have the disadvantages of low flexibility and excessive waste.

Alternatively, digital microfluidics (DMF) chips are becoming a powerful approach to compensate for the disadvantages of continuous-flow microfluidics [10,11,12]. Based on the electrowetting-on-dielectric (EWOD) principle, the DMF relies on the electrode array which is capable of generating electrical potential on the electrodes, then the nanoliter or microliter droplets can be freely and high-accuracy manipulated (e.g., mixing, splitting, and transfer) in two dimensions by the calculating control commands [13,14,15]. The emergence of DMF provides a new research method and tool for nucleic acid automation and rapid analysis. In recent years, digital microfluidics has been widely used in nucleic acid extraction and purification, nucleic acid amplification, pyrosequencing, single nucleotide polymorphism analysis, and other areas [16,17,18,19,20]. For DNA or RNA extraction, obtaining sufficient and pure nucleic acids from raw biological samples is the key to subsequent analysis. Nucleic acid extraction and purification generally include repetitive sampling, mixing, and washing processes; thus, it is especially suitable for the DMF system. Sista et al. used DMF to isolate a pure genome from human whole blood samples [21], Hung et al. also developed a rapid nucleic acid extraction technology based on DMF, and the extraction process was completed at room temperature [22]. PCR is one of the most widely used in vitro nucleic acid fragment amplification technologies for nucleic acid amplification. However, the PCR process requires at least two temperatures to achieve amplification, which will undoubtedly increase the complexity of the DMF system and is not conducive to realizing on-site real-time detection [23]. Therefore, to make better application of the advantages of the DMF system, constant temperature amplification technology has attracted the attention of researchers. This technology only needs a constant temperature zone to achieve the amplification of nucleic acid sequences, and the temperature requirements are not particularly strict, which significantly simplifies the complexity of the temperature control model of the DMF system. Coelho et al. verified the feasibility of LAMP amplification on DMF for the first time [24]. Wan et al. developed DMF-LAMP based on molecular beacons and used a fluorescence microscope to record fluorescence in real-time [20,25]. Therefore, DMF technology has the characteristics of miniaturization of biochemical reactions, allowing low test dose input, fast heat transfer, and highly reconfigurable droplet control, making it a promising candidate for the point-of-care testing (POCT).

Therefore, based on the previous research foundation and the issue that needs to be solved, we introduced an all-in-one nucleic acid detection system based on DMF technology which integrates automatic nucleic acid extraction, rapid nucleic acid amplification, and real-time amplification product detection in sequence. The system combined the magnetic model designed according to the DMF chip, realizing the on-chip nucleic acid extraction process based on the magnetic beads method. Moreover, the system also has heater and optical signal detection models for nucleic acid amplification and real-time result amplification result detection. Thus, the application of the DMF chip in this system can realize the advantages of automatic droplet control, combined with the matching functional modules, and then realize an automated all-in-one nucleic acid extraction and molecular diagnostic system.

## 2. Materials and Methods

### 2.1. Materials and Reagents

Tetronic 90R4 and green dye 5-Carboxyfluorescein (5-FAM) were purchased from Sigma–Aldrich (Product No. 86826). CYTOP were purchased from AGC Chemicals Company, which is a kind of commonly used hydrophobic layer material. Silicone oil (2 cst) was purchased from Dow Corporate. Deionized (DI) water (18.2 MΩ) was supplied by a Milli-Q water purification system. The qPCR reactive enzyme premix was purchased from Invitrogen (Platinum^®^ SYBR^®^ Green qPCR SuperMix-UDG with ROX). LAMP positive control reagents were purchased from Loopamp^®^. LAMP reagents for SARS-CoV-2 nucleic acid detection were developed in our own laboratory. The forward and backward primers for qPCR and LAMP primer sets are shown in Table 1 and were all produced by Sangon Biotech (Shanghai, China). The new coronavirus RNA standard was purchased from Shanghai Institute of Metrology and Testing Technology (the reference virus genome sequence is GenBank No. MT027064.1, the gene coordinate N is the full length of the gene, and the concentration is 10^5^ copies/μL).

### 2.2. The Off and On-Chip Nucleic Acid Extraction

Nucleic acid extraction was performed as follows. We used the same reagent ratios and test procedures for on-chip and off-chip experiments. A total of 5 µL of the biological sample to be extracted was added to 0.5 µL of protease K, 0.5 µL of extraction magnetic beads, and 18 µL of lysate (RM101-AB). These specimens were mixed on or off the chip, and the waste solution containing nontarget products was removed by a magnetic suction module. MBs were washed with 24 µL of washing buffer No. 1 (The Washing liquid RM101-AD) and then 24 µL of washing buffer No.2 (The Floating liquid RM101-AE). Each washing step requires the magnetically module under the DMF chip to cooperate. After each washing, the magnetic module needs to be raised to stick to the bottom of the DMF chip to fix MBs, and then the waste liquid is driven by electrodes to move the waste liquid to the waste area. Finally, 6 µL of elution reagent (RM101-AF) was used for elution to obtain the final nucleic acid product. The magnetic bead nucleic acid extraction kit was purchased from Vazyme Biotech Co. Ltd. (Nanjing, China, Product No. RM101). The whole on-chip nucleic acid extraction process is shown in supporting information Video S1. For product verification after extraction, we chose the qPCR method. The test method was in accordance with the kit instruction, and the test reaction system was 5 µL. The commercial qPCR instrument used for off-chip qPCR and LAMP test was PCRmax Eco48 (PCR max, UK). 

### 2.3. Design of the Digital Microfluidics

**Figure 1:**
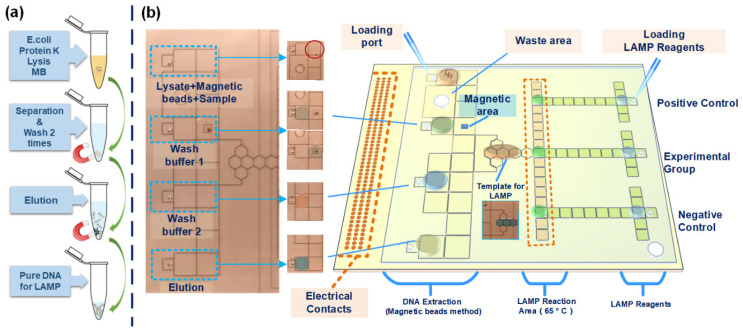
(**a**) Flow chart of off-chip DNA extraction, (**b**) Schematic diagram of the all-in-one on-chip process.

Figure 1a shows the process of the conventional off-chip magnetic bead (MB) method for nucleic acid extraction, which includes cell lysis, separation, washing, and elution. After obtaining the pure nucleic acid sample, we added the matching amplification reaction reagent to it and transferred it to the nucleic acid amplification machine for the corresponding nucleic acid amplification reaction. At the same time, the real-time fluorescence signal of the amplification product was collected to judge the amplification results. According to the off-chip process of the MB method and nucleic acid amplification, the amplification method we applied is the LAMP method, and we designed our DMF chip, which includes three parts (DNA extraction, LAMP reaction area, and reagent of LAMP storage area), as shown in Figure 1b. We set four loading ports for the DNA extraction part, the four injection ports are used for the lysis solution containing sample and MB, wash buffer 1, wash buffer 2, and elution, respectively. Before the test, we need to add the above four solutions to the four liquid storage areas in the DNA extraction area of the chip according to the preset volume. Next, we activate the DNA extraction pathway pre-edited on the upper computer and initiate the automatic program, which includes the pre-heating program of the heater. Then, according to the pre-edited pathway, after the DNA extraction program the pure DNA or RNA template was split and transferred to approximately 1 µL (concentration of DNA was 8 ng/µL) of the LAMP reaction area as a sample of the experimental group (the template from the on-chip DNA extraction process), and the positive, negative, and LAMP reagents were simultaneously transferred to the reaction area. Finally, the heater module has already started by the pre-edited program for the LAMP reaction. At the same time, the optical detection module also starts to collect and record the change in the reaction samples’ fluorescence signal. The detection module uses a photon-counting photomultiplier tube (PMT), the detection signal is transmitted to the PMT through multi-mode fiber, and the fiber probe is assembled with a three-axis system through a customized buckle and placed vertically above the DMF system.

### 2.4. Fabrication of the DMF Chip

**Figure 2:**
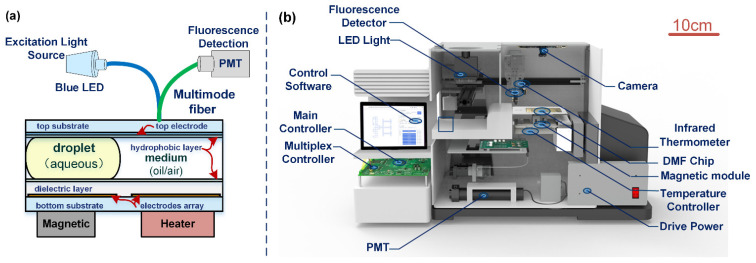
(**a**) Schematics of EWOD all-in-one system, (**b**) Structure diagram of all-in-one system.

In this research, we applied EWOD to manipulate the reaction reagents or biological samples. EWOD has been developed as a driving force to manipulate droplets in parallel plate devices of DMF. As shown in Figure 2a, the DMF system consists of the top substrate, droplet, bottom substrate, magnetic module, and heater module. The DMF chip comprises a printed circuit board (PCB) coated with polyethene (PE) film as the bottom substrate and Indium tin oxide (ITO) glass as the top substrate, both of which are coated with CYTOP (6% (*w*/*w*) in FC-43) as the hydrophobic layer. Two substrates are separated by the Poly tetra fluoroethylene (PTFE) spacer, typically 600 µm. The space between the two substrates is filled with silicone oil (2 cst), which acts as a medium for reagent movement [14]. The overall structure of the DMF chip and a photo of the object are shown in Figure S1 in Supplementary Material.

### 2.5. Development of the Peripheral

As shown in Figure 2b, three high-voltage solid-state multiplexers (HV507 from microchips) were used to provide the sequenced voltage signals for the electrode array for the electronics system. The switching unit and a customized power source were controlled by a microcontroller (STM32H7 from STMicroelectronics). Optical signal detection was realized by a photocounting photomultiplier tube (PMT) module (H11123, Hamamatsu, Japan) as the fluorescence signal acquisition probe, the excitation light was an LED of 470 nm (M470L4, Thorlabs, USA), and the optical signal and extraction light were transmitted through the reflection probe with a round leg multimode fiber (RP22, Thorlabs, USA). The optical fiber probe is controlled by a three-axis mechanical arm, which can realize on-chip multi-point scanning. The magnetic module of the system is realized by cylinder neodymium magnets (diameter of 3 mm and height of 5 mm), which are connected to the steering gear. The software can be used for up- and down-control, and then the relevant operations in the magnetic bead extraction process can be realized. The whole system is about 40 cm × 25 cm × 30 cm in length, width, and height. ACXEL Tech Ltd. developed DMF software for droplet manipulation, which can preprogram the movement path of the droplet, and the software can also control the optical detection, heater, and magnetic modules.

## 3. Results

### 3.1. Validation of the Nucleic Acid Extraction

**Figure 3:**
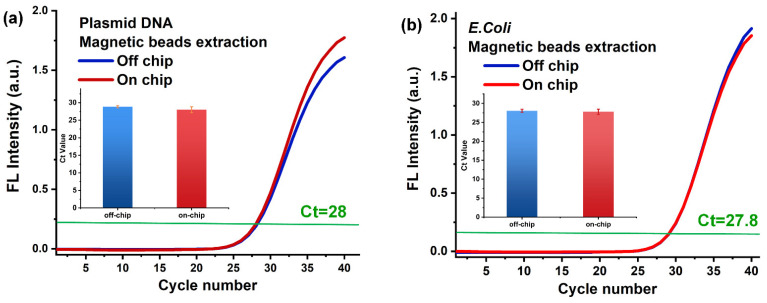
The qPCR results of on-chip and off-chip DNA extraction from the (**a**) Plasmid DNA and (**b**) *E. coli*.

In this research, we mainly applied the MB method for DNA or RNA extraction. We first performed a comparison experiment of on-chip and off-chip DNA extraction process. The extraction results were confirmed by qPCR method. First, we applied pure DNA plasmid samples to perform the nuclide acid extraction experiments on- and off-chip. Then, we conducted qPCR tests on both on-chip and off-chip DNA products that went through the same extraction process. Then, we conducted 40 cycles of qPCR tests on both on-chip and off-chip DNA products that went through the same on-chip extraction process, and the result is shown in Figure 3a. By comparing the on- and off-chip amplification curves, it is determined that their Ct values are almost the same, both of them are around 28, which means that the on- and off-chip DNA extraction quality is almost the same. The histogram figure in the figure measured the statistical results of repeated tests for 4 times, and the results showed good repeatability. The average Ct value of off-chip was 28.8, and that of on-chip was 28. To further confirm that the effect of on-chip nucleic acid extraction is comparable to that of off-chip extraction, we also performed on-chip and off-chip nucleic acid extraction experiments using real biological samples, which were *E. coli* samples, and the results are shown in Figure 3b. The Ct values of the on- and off-chip are almost the same. The average Ct value of off-chip was 28, and that of on-chip was 27.8. The qPCR amplification curves of on-Chip and off-chip DNA extracts repeated for 4 times are shown in Figure S2. 

**Figure 4:**
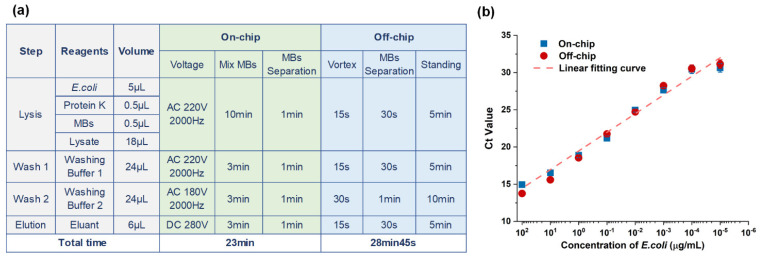
(**a**) Reaction conditions of on-chip and off-chip nucleic acid extraction, and (**b**) comparison of on-chip and off-chip nucleic acid extraction qPCR characterization results of different concentrations of *E. coli*.

Specific on-chip and off-chip reaction conditions are shown in Figure 4a. The entire on-chip extraction time is only 23 min, but the manual operation off-chip takes nearly half an hour. For the off-chip process, the standing time here refers to the stationary time of the sample on the magnetic frame. In order to better collect the magnetic beads suspended in the liquid at the bottom of the magnetic frame, so as to obtain the most target products, we usually need a longer incubation time. The separation time of magnetic beads includes the time when we manually extract supernatant and clean the surface of magnetic beads. Additionally, we did the same process on the chip, and literally pressed the drop into a pie shape. The contact area of the magnetic absorption module below our chip can match the footprint of the droplet, which greatly improves the absorption and aggregation efficiency of magnetic beads. At the same time, each step on the chip can be seamless automatic connection, thus saving part of the time of manual operation. Therefore, our whole process on the chip takes less time than the process off-chip. By comparing the abovementioned results of on-chip and off-chip DNA extraction experiments between plasmid DNA and *E. coli*, it is confirmed that on-chip DNA can achieve almost the same effect as off-chip DNA extraction, and the extracted products can be amplified in the following reaction.

To further confirm that the effect of on-chip MB extraction is equivalent to that of off-chip MB extraction, we also conducted a comparative experiment of on- and qPCR-characterized off-chip MB DNA extraction for *E. coli* samples with a linear concentration gradient and the extracted DNA samples. Based on previous studies, we selected different driving voltages for different reaction reagents. According to the reaction conditions shown in Figure 4a, we conducted off- and on-chip DNA extraction experiments, and the sample that was applied was E. coli. We set up eight sets of reactions according to the concentration gradient and repeated each extraction reaction three times. Finally, qPCR was performed using the extracted DNA sample as the template, and the result is shown in Figure 4b. After fitting the eight groups of Ct values of on- and off-chip reactions, we find that the on- and off-chip reaction results have good linearity. The error bar of each group of reactions is relatively small, which confirms that the on-chip reaction has good repeatability. The extraction effect is the same as the off-chip reaction effect, confirming that the on-chip DNA extraction function can be applied to nucleic acid extraction experiments and subsequent amplification detection reactions.

### 3.2. Validation of the LAMP Reaction Function

After verifying the nucleic acid extraction function of the whole system, we need to verify the thermostatic amplification reaction function of the whole system. For the LAMP reaction part, we first verify the function of the temperature control module and the optical detection module. We placed a PTC resistor inside the fixed temperature zone (the 65 °C LAMP reaction area) to detect the temperature of the heating module, placed a temperature sensor above the bottom structure to detect the temperature of the droplet and oil, and used Kalman filtering, temperature control PID, and other algorithms to control the temperature accuracy to ±0.5 °C. 

**Figure 5:**
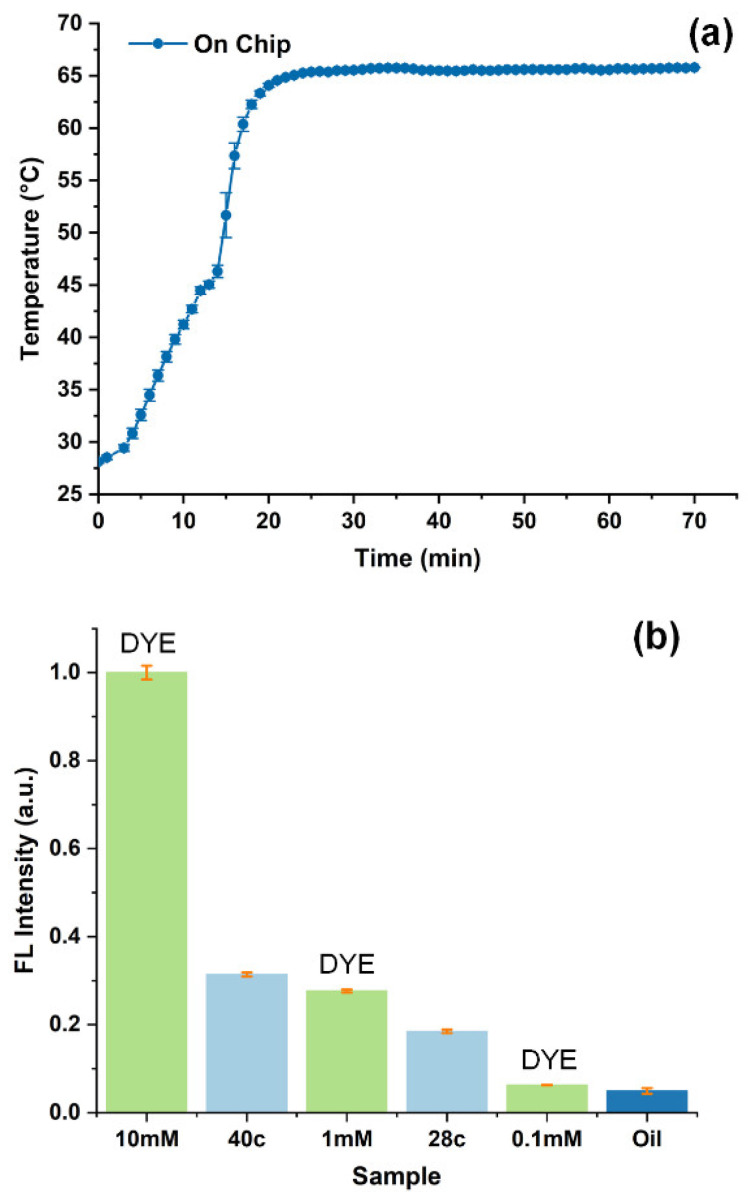
(**a**) The temperature curve of the temperature controller, (**b**) the detection resolution test of optical detection module.

As shown in Figure 5a, after testing the temperature control module ten times, the test result shows that when the ambient temperature is 27 °C, the reaction temperature of LAMP can reach 65 °C after 22 min. Moreover, for the optical detection model, different concentrations of green fluorescent FAM dye and products of 28 cycles (28c) and 40 cycles (40c) of qPCR were used to verify the function of the optical detection module, and the test results are shown in Figure 5b. The lowest dye concentration we could distinguish was 0.1 mM, and we could also distinguish 28 cycles of qPCR products, which was the Ct value in our abovementioned experiment (Figure 3b).

**Figure 6:**
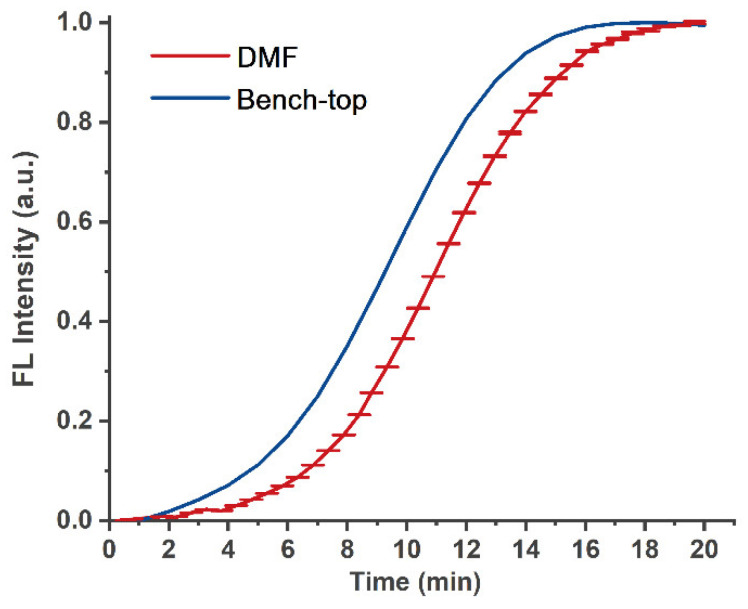
On-chip and off-chip amplification curves of LAMP amplification reaction.

Considering the abovementioned results, we selected positive standard products from commercial kits to verify the LAMP amplification reaction function on the DMF chip. After 1 µL of reagents containing positive templates and primers were effectively mixed with 1 µL of LAMP reaction mixed enzyme reagents in the LAMP reaction area on the chip, the temperature control and optical detection modules were turned on to collect real-time fluorescence signals for the LAMP reaction. At the same time, positive control reagents with the same reaction concentration were set up in a commercial qPCR machine, which set just one reaction temperature, 65 °C, and 40 cycles. Due to the limitation of the reaction system of the commercial qPCR instrument, the LAMP reaction system of 5 µL was used in the off-chip experiment. The real-time fluorescence signal was collected by the qPCR machine and DMF system, the positive threshold was reached at approximately 18 min, as shown in Figure 6. For the signal collection on the chip, in order to ensure the accurate determination of the collected data, we collected data three times in each cycle, and then taking the average value of the three times, as shown in Figure 6, it can be seen that the error bar is relatively small. The LAMP reaction usually judges whether the time taken to reach the threshold point is within the specified reaction time.; thus, the on-chip real-time LAMP reaction achieved almost the same test results as a commercial machine.

### 3.3. Functional Verification of All-in-One DMF System in SARS-CoV-2 Detection

**Figure 7:**
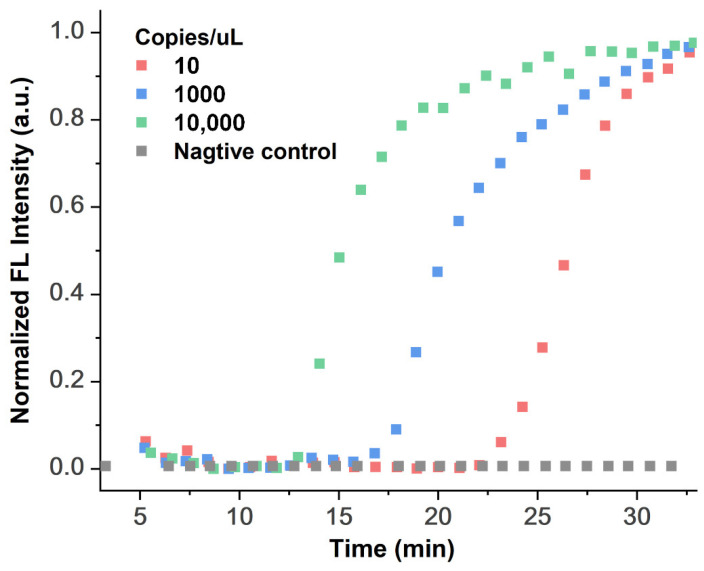
Real-time LAMP fluorescence curve for detecting the RNA of pseudo-coronavirus on the DMF chip.

The on-chip all-in-one process test was evaluated by SARS-CoV-2 nucleic acid reference materials, which are in vitro transcribed RNAs. Through the method of diluting RNA samples step by step, we selected 10, 1000, and 10,000 copies of RNA samples per microliter to perform the on-chip full-process and automated nucleic acid detection experiment, which includes MB method-based RNA extraction, LAMP amplification reaction, and real-time LAMP amplification signal collection. Because it is not possible to use real novel coronavirus biological samples for RNA extraction, diluted SARS-CoV-2 nucleic acid reference materials were selected for functional verification experiments in accordance with the RNA extraction process of the magnetic bead method. A total of 5 µL of RNA sample after the magnetic bead extraction process was evenly separated into two 1 µL droplets on chip as the template of the experimental group, while the template of the negative control group was free of nucleic acids. Then, 2 droplets of the experimental group and 1 droplet of DI water for the negative control group were moved to the LAMP reaction area. Finally, 3 droplets of the LAMP master mix reaction solution were also moved to the LAMP reaction area for on-chip mixing with 2 droplet of template and 1 droplet of DI water. Then, the temperature control module and optical detection module were used. The thermal cycling conditions were 40 cycles of reaction at 65 °C; each cycle was 1 min; the fluorescence signal was collected every 1 min, and the real-time amplification curve obtained is shown in Figure 7. During the LAMP reaction, at the electrode where the LAMP reaction droplets are located, we maintain the droplets in a fixed reaction area by applying a lower voltage (150 V at 50 kHz) to avoid mutual interference between the droplets. Thus, we only need to manually add samples and other extraction and detection reagents to the corresponding positions for the whole detection process. We tested three SARS-CoV-2 nucleic acid reference materials at different concentrations for all-in-one process on-chip assay reactions, with a limit of detection of 10 copies/µL. The subsequent nucleic acid extraction and detection processes are automatically completed following the present path and matching magnetic and heating procedures, a “sample to answer” in approximately 1 h, which includes 23 min of RNA extraction and 35 min for the LAMP reaction.

## 4. Conclusions

We developed an all-in-one DMF system that realized automatic MB-based nucleic acid extraction, LAMP amplification, and real-time amplification product detection. By comparing the on-chip and off-chip DNA extraction experiments of pure DNA plasmid samples and E. coli samples, we verified that the efficiency of on-chip DNA extraction was identical to that of off-chip DNA extraction, and the process was automated and controllable in a reduced amount of time. We also compared the on-chip LAMP amplification reaction with the off-chip reaction, and the results for the two experimental groups were similar under the same reaction conditions. Finally, we used RNA from SARS-CoV-2 nucleic acid reference materials as samples and performed on-chip full-flow, including auto-mated nucleic acid extraction, LAMP amplification, and real-time fluorescence signal detection. The detection results of SARS-CoV-2 nucleic acid reference materials have potential value in detecting DNA or RNA in clinical samples for the rapid diagnosis of infectious diseases, especially with the current outbreak of a global pandemic.

## Figures and Tables

**Table 1:** Primer list of qPCR and LAMP reaction.

**qPCR**	F: CATGCCGCGTGTATGAAGAA
	R: GGGTAACGTCAATGAGCAAA
**LAMP**	F3: CCAGAATGGAGAACGCAGTG
	B3: CCGTCACCACCACGAATT
	FIP: AGCGGTGAACCAAGACGCAGGGCGCGATCAAAACAACG
	BIP: AATTCCCTCGAGGACAAGGCGAGCTCTTCGGTAGTAGCCAA
	LF: TTATTGGGTAAACCTTGGGGC
	LB: TTCCAATTAACACCAATAGCAGTCC

## Data Availability

Not applicable.

## Supplementary Materials

The following supporting information can be downloaded at https://www.mdpi.com/article/10.3390/bios12050324/s1, Figure S1. The overall structure of the DMF chip and the photo of the object. Figure S2. The qPCR amplification curves of on-Chip and off-chip DNA extracts repeated for 4 times. Video S1: The whole on-chip nucleic acid extraction process. Video S2: Animation of droplet distribution of nucleic acid product after extraction.
