# Supplementary material for: All-in-One Digital Microfluidics System for Molecular Diagnosis with Loop-Mediated Isothermal Amplification
Source: Biosensors (Basel). 2022 May 11;12(5):324. doi: 10.3390/bios12050324 (PMC9138765; doi:10.3390/bios12050324)
Supplement: Supplementary file 1 [file biosensors-12-00324-s001.zip › biosensors-1711340-Supplementary Materails.pdf]

Electronic Supporting Information.

All-in-one Digital Microfluidics System for Molecular Diagnosis with Loop-mediated Isothermal Amplification

Siyi Hu[1], Yuhan Jie[2], Kai Jin[1], Yifan Zhang[1], Tianjie Guo[1], Qi Huang[1], Qian Mei[1], Fuqiang Ma[*1] and Hanbin Ma[*1,2]

1. CAS Key Laboratory of Bio-medical Diagnostics, Suzhou Institute of Biomedical Engineering and Technology, Chinese Academy of Sciences, No.88 Keling Road, Suzhou, Jiangsu, 215163, P.R. China.

2. Guangdong ACXEL Micro & Nano Tech Co., Ltd, Guangdong province, 528000, P.R.China

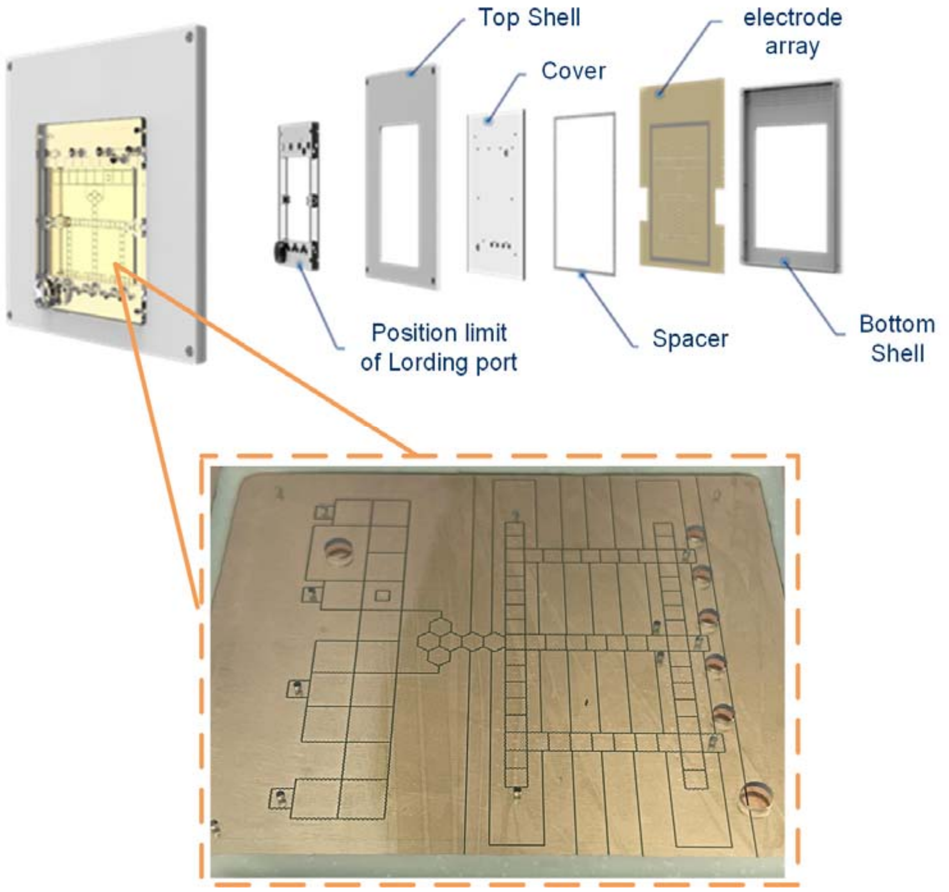

Figure S1. The overall structure of the DMF chip and the photo of the object.

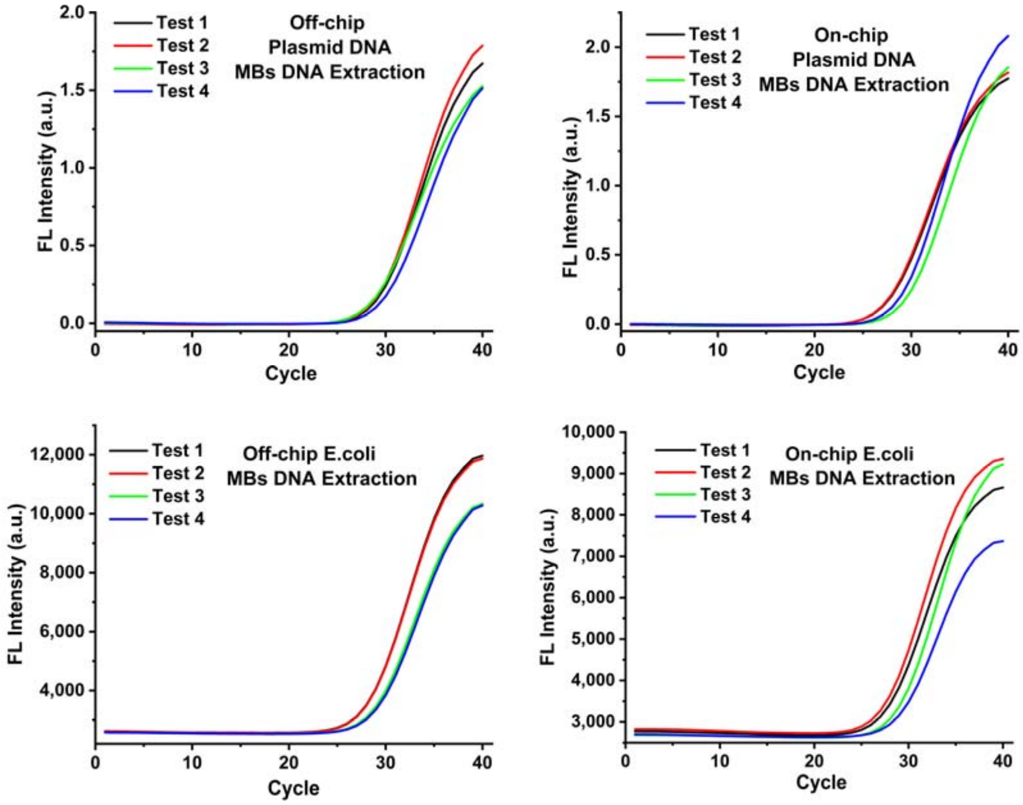

Figure S2. The qPCR amplification curves of on-Chip and off-chip DNA extracts repeated for 4 times
